# A Case in Practice

**Published:** 1879-04

**Authors:** 


### A CASE IN PRACTICE.

Editor Dental Register:—Four weeks ago, S. D., age 26, called to have me fill some teeth for him; two were approximate fillings, in the first and second left superior molars, the cavities were large, but not very sensitive. At the patient's request I filled them with amalgam, after having prepared the cavities in the most thorough manner. A week after, he called to have the fillings finished up thoroughly; the filling in the anterior surface of second molar was found to be all right, but in the one occupying the distal surface of first molar I found it loose. I at first attributed it to the contraction of the metal, and went to work to remove the filling, which operation being completed, judge my surprise to find in the centre of the cavity, an elevation the size of a mustard seed, exceedingly sensitive, so much so that the slightest touch with a piece of spunk, caused unbearable pain. I applied the rubber dam, wiped the cavity with carbolic acid, and inserted an amalgam filling, which has proved successful thus far, the tooth feeling all right at this time. As regards the nature of that elevation, my theory is that, in excavating the cavity I probably cut through a fissure of the pulp chamber, that had run out farther toward the periphery of the tooth than the rest of the pulp chamber, and in making said cut, wounded the pulp, which being in a perfectly healthy condition, set about to repair the injury I had done. The pulp throwing out lymph highly charged with nerve tissue material, which having calcified, still retained the high degree of sensibility peculiar to this structure. And I attribute the loosening of the filling, to the deposition of said material. Is my diagnosis right?

